# Determination of skin-insect repellent icaridin and DEET in human urine using solid-phase extraction and liquid chromatography with tandem mass spectrometry and its application to a sample of Japanese adults

**DOI:** 10.1265/ehpm.24-00220

**Published:** 2025-03-20

**Authors:** Nanami Nishihara, Tomohiko Isobe, Mai Takagi, Toshiki Tajima, Yugo Kitahara, Mai Hayashi, Isao Saito, Satoru Watanabe, Miyuki Iwai-Shimada, Jun Ueyama

**Affiliations:** 1 Department of Biomolecular Sciences, Field of Omics Health Sciences, Nagoya University Graduate School of Medicine, 1-1-20 Daiko-minami, Higashi-ku, Nagoya 461-8673, Japan; 2 Health and Environmental Risk Division, National Institute for Environmental Studies, 16-2 Onogawa, Tsukuba, Ibaraki 305-8506, Japan

**Keywords:** Insect repellent, Icaridin, DEET, Human biomonitoring, LC-MS/MS

## Abstract

**Background:**

Icaridin and DEET are common insect repellents widely used on human skin and clothing (skin-insect repellents [skin-IR]) to repel common pests, such as mosquitoes and biting flies. Novel analytical methods for urinary skin-IR exposure biomarkers that can be effectively applied in epidemiological studies and provide strong evidence related to risk assessment associated with daily exposure are required. In this study, we aimed to develop a method for analyzing the concentrations of icaridin, DEET, and two DEET metabolites *N*,*N*-diethyl-3-(hydroxymethyl) benzamide and 3-(diethylcarbamoyl) benzoic acid in human urine.

**Methods:**

In this analysis, after formic acid-induced acidification of the urine sample, exposure biomarkers were extracted using solid-phase extraction composed of a modified polystyrenedivinylbenzene polymer for reversed phase (hydrophobic) retention. Subsequently, high-performance liquid chromatography-tandem mass spectrometry was performed within 10 min for a separation analysis. The present method was applied to five Japanese adults (aged 20–43 years) who used icaridin or DEET-containing products within a week.

**Results:**

Limits of detection were 0.06–0.11 µg/L. Extraction recoveries were 74%–88%. The intraday and interday variations were 1.5–17.5 and 0.9–15.8% relative standard deviation, respectively. All exposure biomarkers were successfully detected in all five adults. Urinary concentrations of exposure biomarkers reached their maximum values within 15 h after starting to use skin-IR.

**Conclusions:**

This method was successful in measuring urinary exposure biomarkers of skin-IR, including icaridin and DEET. Moreover, this study presents the first application of biomonitoring of urinary icaridin concentrations after using a commercial product.

**Supplementary information:**

The online version contains supplementary material available at https://doi.org/10.1265/ehpm.24-00220.

## Graphical Abstract

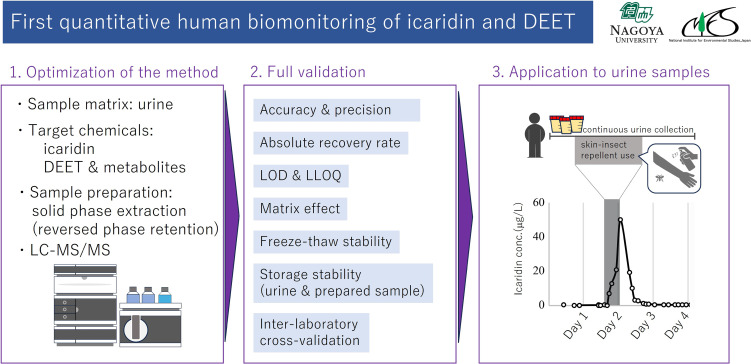

## Introduction

The direct application of skin-insect repellents (hereafter called skin-IR) is an effective way to protect humans from disease-transmitting arthropods, including mosquitoes, sandflies, ticks, fleas, and mites [1–6]. Currently, skin-IR products are frequently applied on adults and children, mostly during outdoor activities, such as farmwork, playing outdoors, and hiking. The demand and use of skin-IR products are expected to grow owing to global warming, which promotes population growth in disease-transmitting arthropods. Icaridin (IUPAC name: *sec*-butyl 2-(2-hydroxyethyl) piperidine-1-carboxylate; WHO designation) and DEET (IUPAC name: *N*,*N*-diethyl-3-methylbenzamide) are active ingredients in many skin-IR products. DEET has been widely used for more than six decades [7]. Icaridin was developed by Bayer Corporation and was commercialized around the world after DEET (Europe since 1998, the United States since 2005, and Japan since 2016) [8, 9]. Unlike DEET, icaridin is odorless, non-greasy, does not dissolve plastics or other synthetics, and does not influence the sun protection factor or percutaneous absorption when applied with sunscreen [10]. Furthermore, as icaridin is skin-friendly, even for young children, it may be used more frequently in the future [11].

Previously, a 2-year feeding experiment using rats, mice, and dogs revealed a reduction in body weight and food consumption induced by high doses of DEET in female rats and a slight increase in serum cholesterol levels [12]. A few previous reports demonstrated that high levels of exposure to DEET potentially cause undesirable health hazards, such as central nervous system toxicity, seizures, and encephalopathy in children [13–15]. However, due to the limited quantitative assessments of exposure levels in these reports, estimating the potential human health risks associated with DEET remains unclear. More recently, Cui et al (2022) suggested that a high DEET exposure level estimated by urine analysis is positively associated with the prevalence of obesity, including abdominal obesity, in the general adult population [16]. The toxicity of icaridin has been explored mainly based on laboratory experiments. The possible adverse effects observed in experimental animal experiments are liver necrosis and hyaline degeneration in the kidney, with subchronic dermal and subacute dietary toxicity. Furthermore, *in vitro* experiments revealed that icaridin administration led to chromosomal effects, including an increased incidence of cells exhibiting chromatid or chromosomal aberrations [17]. The non-observed adverse effect level (NOAEL) for dermal chronic toxicity of icaridin was reported to be 200 mg/kg BW/day (cystic degeneration of the liver) [18]. However, adverse effects of icaridin exposure on human health in real life have rarely been reported. Customers may apply skin-IR products in a manner inconsistent with label statements [19, 20]; moreover, ingredient exposure and absorption levels may vary with different commercial products, areas of application on the skin, and absorption rates at different application sites among individuals [21]. Thus, there is an urgent need to assess the icaridin exposure at the individual level, which can play a pivotal role in risk assessment using epidemiological study.

The development of novel methods for the simultaneous determination of skin-IR exposure biomarkers applicable to human urine is required to provide strong evidence for exposure and risk assessments. An experimental study [11] showed that icaridin may be excreted in the urine and serve as a possible exposure biomarker in humans. Although two previously published reports have claimed that urinary icaridin was detected using non-targeted screening method without standard reagents [22, 23], there are no quantitative data on icaridin exposure using biomonitoring, which is attributable to the scarcity of analytical methods established for urinary icaridin measurement. However, DEET has been used worldwide for several decades; thus, biomonitoring using DEET and its metabolites as exposure biomarkers has been extensively studied [24–27]. Kuklenyik et al. (2013) [25] successfully developed an on-line solid-phase extraction (SPE)-high-performance liquid chromatography-tandem mass spectrometry (LC-MS/MS) method for quantification of urinary DEET and its oxidative metabolites. Their study was motivated by the fact that off-line SPE requires considerable sample handling (e.g., evaporation and reconstitution of SPE urine extract) and can be labor-intensive. However, the off-line SPE method has relatively unlimited flexibility, making it a suitable procedure for trace enrichment analysis in diverse analytical applications in many laboratories.

**Fig. 1:**
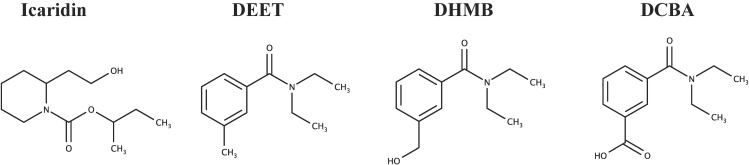
Chemical structures of icaridin, DEET, and their oxidative metabolites.

Therefore, in this study, we aimed to develop an off-line SPE LC-MS/MS measurement method for skin-IR exposure biomarkers in urine that can be applied in epidemiological studies. Specifically, the urinary biomarkers icaridin, DEET, and the ring methyl oxidation metabolites *N*,*N*-diethyl-3-(hydroxymethyl)benzamide (DHMB) and 3-(diethylcarbamoyl) benzoic acid (DCBA) [24] (Fig. 1), were targeted. The method was verified using urine samples obtained from five Japanese adults (20–43 years old) who were previously exposed to icaridin or DEET-containing products.

## Materials and methods

### Chemicals and reagents

A standard reagent of icaridin (95% purity) was purchased from Cayman Chemical Co. (Ann Arbor, MI, USA). DEET (98.0% purity) was obtained from Tokyo Chemical Industry Co., Ltd. (Tokyo, Japan). DHMB, DCBA, icaridin-d_3_ (97% purity), and DEET-d_10_ (98% purity) were obtained from Toronto Research Chemicals Inc. (Toronto, ON, Canada). As internal standard (I.S.), icaridin-d_3_ and DEET-d_10_ were used for quantification of icaridin and DEET, DHMB, and DCBA, respectively. LC-MS grade ultrapure water, methanol, acetonitrile, and formic acid (99% purity) were obtained from FUJIFILM Wako Pure Chemical Corp. (Osaka, Japan). Distilled water with 0.5 × 10^−4^ S/m grade was prepared using Auto Still^®^ (Yamato Scientific Co., Ltd., Tokyo, Japan) in our laboratory. β-glucuronidase/arylsulfatase from *Helix pomatia* (5.5 U/mL) was obtained from Roche Diagnostics GmbH (Mannheim, Germany). Potassium phosphate buffer was purchased from Merck (Darmstadt, Germany). The EVOLUTE^®^ EXPRESS ABN 30 mg Plate (Biotage, Uppsala, Sweden), a water-wettable polymeric sorbent with non-polar interactions SPE product, was used to extract skin-IR exposure biomarkers from urine samples. SPE was performed using Extrahera^TM^ (Biotage, Uppsala, Sweden), an automation system with plate formats.

### Pooled urine samples and standard solutions

Pooled urine, prepared using samples collected from five healthy volunteers, was used for all optimization studies, including the optimization of the SPE procedure, calibration curve preparation, and the other validation assays. Standard solutions of icaridin, icaridin-d_3_, and DEET-d_10_ were dissolved in acetonitrile to obtain a concentration of 1 mg/mL. DEET, DHMB, and DCBA were dissolved in water to a concentration of 1 mg/mL. These standard solutions were diluted with acetonitrile at a concentration of 100 µg/mL (working solution) and stored at −40 °C. Icaridin-d_3_ and DEET-d_10_ solutions were stored at −40 °C in the dark.

### Sample preparation procedure

**Fig. 2:**
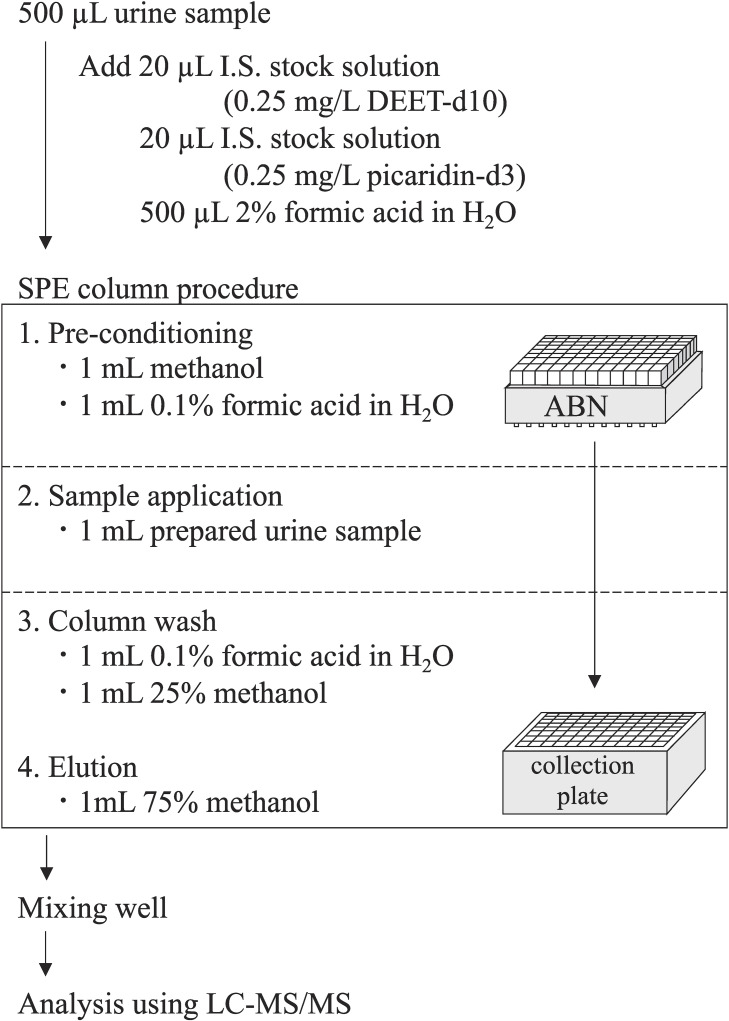
Established method for the analysis of urinary skin-IR exposure biomarkers.

The sample preparation procedure for measuring urinary analytes is schematically shown in Fig. 2. Briefly, 500 µL urine sample was added to a 96-well plate (individual well capacity 2 mL), and 20 µL of each I.S. (0.25 mg/L icaridin-d_3_ and DEET-d_10_) and 500 µL of 2% formic acid were added. Mixing was carried out by pipetting.

The prepared urine sample was subjected to SPE using the automation system Extrahera^TM^, equipped with a nitrogen positive pressure unit, following the manufacturer’s instructions. Preconditioning of ABN cartridge was performed using 1 mL methanol, followed by equilibration with 1 mL H_2_O containing 0.1% formic acid. Subsequently, 1 mL prepared urine sample was loaded into the ABN cartridge. After passing the sample through ABN cartridge, it was washed with 1 mL of 0.1% formic acid, followed by washing with 1 mL of methanol:H_2_O (1:3, *v*/*v*). Target compounds were eluted with 1 mL of methanol:H_2_O (3:1, *v*/*v*). Finally, eluents were analyzed using LC-MS/MS.

### Chromatography and mass spectrometry conditions

LC-MS/MS analysis was conducted using an Agilent 1260 Infinity Binary LC system coupled with an Agilent 6430 triple quadrupole mass spectrometer (Agilent Technologies, Inc., CA, USA). The following LC operating conditions were adopted for the analysis: InertSustain AQ-C18 (150 mm × 2.1 mm i.d., 3 µm particle size; GL Sciences, Tokyo, Japan) LC column, H_2_O containing 0.1% of formic acid as mobile phase A, acetonitrile as mobile phase B; gradient condition of mobile phase B as 40–90% (0–6 min)-90% (6–9 min)-40% (9–13 min), total flow rate of 0.2 mL/min, with a 13 min run time per sample, and 8 µL injection volume. Ionization was performed in positive ion mode followed by multiple reaction monitoring using MS/MS. The nebulizer gas pressure, source temperature, and gas flow were 50 psi, 300 °C, and 10 L/min, respectively. The capillary voltage was 4000 V (positive mode), and high-purity nitrogen gas (>99.9995%) was used in the collision cell. Table 1 shows optimized multiple reaction monitoring parameters and retention times for icaridin, DEET, DHMB, DCBA, and two I.S. agents. The chromatograph and mass spectrogram data were recorded using the Mass Hunter Software Workstation (Agilent Technologies, Inc., CA, USA).

**Table 1:** Compound-specific mass spectrometer settings and retention times.

**Compound**	**Fragmentor** **(V)**	**Collision energy** **(eV)**	**Precursor ion** **(*m*/** ***z*** **)**	**Product ion** **(*m*/** ***z*** **)**	**Retention time** **(min)**
Icaridin	90	8	230	130 (Q)	6.1
	90	5	230	156 (C)	
	90	2	230	174 (C)	
Icaridin-d3	100	10	233	130 (Q)	6.0
DEET	140	30	192	91 (C)	6.2
	140	19	192	119 (Q)	
DHMB	130	27	208	89 (C)	3.2
	130	17	208	135 (Q)	
DCBA	120	30	222	121 (C)	3.6
	120	20	222	149 (Q)	
DEET-d_10_	140	10	202	119 (Q)	6.2

Q, quantification ion; C, confirmation ion

In addition to InertSustain AQ-C18, the performance of the LC column was compared with that of the following seven other LC columns: Poroshell 120 EC-C18 (75 mm × 2.1 mm i.d., 2.7 µm silica; Agilent, CA, USA), InfinityLab Poroshell 120 CS-C18 (100 mm × 2.1 mm i.d., 2.7 µm silica; Agilent), ZORBAX SB-C18 (50 mm × 2.1 mm i.d., 1.8 µm silica; Agilent), CAPCELL PAK C18 (150 mm × 2.0 mm i.d., 3 µm silica; SHISEIDO, Tokyo, Japan), CAPCELL CORE ADME (150 mm × 2.1 mm i.d., 2.7 µm silica; SHISEIDO), CAPCELL CORE PFP (150 mm × 2.1 mm i.d., 2.7 µm silica; SHISEIDO), and Cadenza CD-C18 (150 mm × 2.0 mm i.d., 3 µm silica; Imtakt, Kyoto, Japan). Of these, the better LC column was selected after comparison with the performance parameter, the height equivalent of one theoretical plate (HETP). HETPs were calculated with multiple reaction monitoring chromatogram concentrations at 40 µg/L each.

### Assay validation

The developed method was validated in terms of matrix effects, precision, extraction recovery, linearity, limit of detection (LOD), and lower limit of quantification (LLOQ).

The matrix effects of exposure biomarkers are presented as the absolute and relative matrix factors following the determination of pooled urine spiked with icaridin, DEET, DHMB, and DCBA at concentrations of 1, 10, 40, and 100 µg/L, respectively, that is, matrix effects were determined through the use of urine extracts and standard solutions. In addition, 1,000 and 10,000 µg/L DCBA were tested considering its higher urinary excretion rate than that of the other DEET exposure biomarkers. The absolute matrix factor was calculated by dividing the peak area of analytes in the urine matrix by that of analyte standard solutions in 75% methanol; the relative matrix factor was calculated by dividing the peak area ratio of exposure biomarkers to I.S. in the urinary matrix by that of exposure biomarker solutions in 75% methanol. Exposure biomarker standard solutions and I.S. solutions were spiked prior to LC-MS/MS analysis procedure. These assays were duplicated.

The intraday precision was examined by determination of pooled urine spiked with icaridin, DEET, DHMB, and DCBA at 0.5, 1, 3, 10, 20, and 40 µg/L (*n* = 6) concentrations of each. For DCBA, concentrations of 200, 2000, and 12,000 µg/L were also considered for the determination of intraday precision. The interday precision was evaluated at concentrations of 0.5, 1, 3, 10, 20, 40, 200, 2000, and 12000 µg/L for five consecutive days. The precision of the method was evaluated by calculating the relative standard deviation (RSD [%]).

Absolute recovery rates of analytes were calculated by a duplicate assay at a concentration of 10 µg/L. The standard solution mixture of exposure biomarkers was spiked at two different points during the measurement procedure—(i) spiking prior to the sample preparation steps and (ii) spiking of samples prepared prior to LC-MS/MS analysis. The total absolute recovery percentage through all the measurement procedures was calculated as the peak area of (ii) to (i).

Calibration curves were constructed by plotting the analyte/I.S. peak area ratios versus the concentrations of 0.5, 1, 3, 10, 20, and 40 µg/L. DCBA concentrations of 200, 2000, and 12000 µg/L were considered. The linearity of the calibration plot provided a slope and intercept, which was considered for determining unknown sample concentrations. Calibration curves with coefficients of determination (*R*^2^) ≥ 0.97 were considered linear. The calibration samples were prepared by spiking standard solutions into the pooled urine.

LOD was defined as a value with a signal-to-noise ratio of 3. LLOQ was defined as a value with a signal-to-noise ratio of 10 and an RSD < 20% for repeated measurements. These values were calculated by the results of analyzing pooled urine samples designed to concentrations around the LOD and LLOQ values using standard solutions.

### Sample stability

Stability of the prepared samples at concentrations of 0.5, 1, 3, 10, and 20 µg/L was tested in triplicate. The prepared sample was stored on an LC autosampler at 4 °C and analyzed at two time points, i.e., at 0 h (immediately) and 48 h. Stability was assessed by comparing the target peak area (absolute area and I.S. ratio) of samples stored for 48 h with that of non-stored samples.

Next, the freeze-thaw stabilities of icaridin, DEET, DHMB, and DCBA present in the urine at concentrations of 0.5, 3, and 20 µg/L were evaluated. Each sample was thawed in tap water for 10 min; after 1 h, the urine samples were placed in a freezer for one day. Samples were analyzed after the third freeze-thaw cycle. Stability was evaluated by comparing concentrations with those of samples that did not undergo the freeze-thaw cycles (*n* = 4).

Storage stability of the analytes present in the urine was estimated using pooled urine samples containing standards spiked at concentrations of 0.5, 3, and 20 µg/L. Urine samples were stored at 4 °C or ambient temperature (25 °C) in the dark for 1 day, 3 days, and 1 week, and their concentrations were measured. The I.S. ratios of stored samples were compared with those of non-stored samples (*n* = 2).

### Application of the developed method to urine samples

All study protocols adhered to the principle of the Declaration of Helsinki. Signed informed consent was obtained from all participants, and the Ethics Committee of the Nagoya University Graduate School of Medicine (approval number: 2020-0187) and the National Institute for Environmental Studies Medical Research Ethics Review Committee (approval number: 2020-002) approved the study protocol. In total, 100 university staff and students at Nagoya University were recruited for this analysis, and their responses to the provided questionnaires were obtained. Urine samples were collected from the participants for five consecutive days, stored at 4 °C, and transferred to the laboratory within 24 h, where the samples were stored at −80 °C prior to analysis. Among the 100 participants, five Japanese adults (20–43 years old) had used icaridin- or DEET-containing products within five days prior to the study. We analyzed the urine of some participants who did not use icaridin or DEET-containing skin-IR products, but the target analytes were not detected, so we only analyzed urine from participants who used the skin-IRs. A total of 155 samples collected from five adults were analyzed to determine the applicability of the developed method. Urine samples were collected over the course of the entire 5-day study period. Participants were allowed to urinate freely without any predetermined schedule.

Moreover, as a preliminary cross-validation assay, a cross-check was conducted using 6 blinded concentration urine obtained from 5 additional healthy volunteers, distinct from the previously mentioned 100 participants and National Institute of Standards and Technology (NIST SRM 3673, Standard Reference Material for Organic Contaminants in Non-Smokers’ Urine). These samples were analyzed by a chemical analysis company located in Osaka prefecture (Lab. A) and Nagoya University, which developed the measurement method (Lab. B). The concentration differences were represented using the percent deviation by the following formula: (A − B)/(average of A and B) * 100. “A” represents the concentration measured in Lab. A, while “B” represents the concentration measured in Lab. B.

### Preliminary test of deconjugation procedure

The deconjugation procedure was assessed using an enzymatic reaction to confirm the conjugation form of the DEET metabolites. Three urine samples that showed high concentrations of urinary DEET were selected from one of the aforementioned five participants who used DEET-containing products. Briefly, the deconjugation procedure was as follows: Urine (250 µL) was diluted twice and 20 µL of DEET-d_10_ solution was added to it. Following this, 15 µL of β-glucuronidase/arylsulfatase from *Helix pomatia* and 0.2 M potassium phosphate buffer (pH 6.8) were added to the sample for deconjugation. After incubation at 37 °C for 17 h, 500 µL of 2% formic acid was added to the sample, which was then subjected to the SPE procedure. The prepared samples were analyzed with LC-MS/MS. The ratio of the target area to the I.S. peak area of the urine samples was compared with or without the deconjugation procedure.

## Results

### Optimization of sample preparation and LC-MS/MS analysis

The aim of our study was to develop an analytical method to quantify icaridin, DEET, and DEET metabolites simultaneously in human urine samples for exposure assessment of skin-IRs. Our novel method is based on simple pretreatment, low-volume solvent usage, no evaporation and reconstitution procedure, minimal off-line sample preparation, and optimal LC-MS/MS analysis (improved separation column and mobile phase).

As shown in Electronic Supplementary Material Table S1, the lower HETP of the analyte chromatogram was obtained using InertSustain AQ-C18. There was no visual problem with any of the peak shapes obtained from the column. Additionally, we confirmed that the mobile phase containing formic acid and acetonitrile was better than that containing acetic acid and methanol regarding their HETPs and peak areas (data not shown). Thus, we selected InertSustain AQ-C18 and water/acetonitrile containing formic acid as one of the optimum separation analysis conditions.

Notably, the washing and elution procedures that involved various percentages of methanol of SPE were optimized. In comparison to icaridin and DEET, DHMB and DCBA were more readily eluted by methanol. The elution behavior varied slightly between DEET metabolites and other target compounds. While a 25% methanol solution could not elute DEET metabolites (less than 10% of the total amount loaded onto the SPE column), a 50% methanol solution successfully eluted approximately 90% of the total DEET metabolites. On the other hand, icaridin and DEET were not eluted with a 50% methanol solution at all (less than 5%). In contrast, more than 85% of the total icaridin and DEET amount loaded onto the SPE column was eluted with 75% methanol solution. Consequently, 25% and 75% methanol solutions were considered optimal solutions for SPE washing and elution, respectively. The results of extracted ion chromatogram of pooled urine spiked with analytes at various concentration ranges (0.5–40 µg/L) showed no interference peaks to the analytes and LLOQ ranging from 0.21 to 0.37 (Table 2). Typical chromatograms are shown in Fig. S2.

**Table 2:** Recovery rate, precision, linearity, LOD, and LLOQ of the analytical procedure.

	**Concentration** **(µg/L urine)**	***n***	**Results**			

			**Icaridin**	**DEET**	**DHMB**	**DCBA**
Absolute recovery rate (%)						
	10		88	74	74	76
Intraday precision (% RSD)						
	0.5	12	10.2	6.9	17.0	13.3
	1	12	6.8	9.7	7.3	9.8
	3	12	4.2	3.8	7.4	5.8
	10	12	3.1	3.3	4.4	3.0
	20	12	1.2	2.6	7.5	6.7
	40	12	1.7	4.0	9.4	5.9
	200	6				4.0
	2,000	6				3.8
	12,000	6				4.4
Interday precision (% RSD)						
	0.5	5	8.3	14.4	15.8	7.6
	1	5	4.8	9.2	11.9	3.1
	3	5	2.1	2.1	6.2	2.8
	10	5	1.4	0.9	8.6	5.9
	20	5	0.9	1.4	5.3	1.9
	40	5	2.0	1.7	10.2	6.7
	100	5				4.6
	1,000	5				7.0
	12,000	5				1.0
Calibration curve						
Slope			0.036	0.050	0.028	0.015
Intercept			−0.009	−0.011	−0.004	0.001
*R*^2^			0.999	0.999	0.999	0.999
LOD (µg/L, S/N = 3)			0.07	0.06	0.10	0.11
LLOQ (µg/L)			0.24	0.21	0.33	0.37

N, number of observations; RSD, relative standard deviation; *R*^2^, coefficient of determination; LOD, limit of detection; LLOQ, lower limit of quantification; S/N, signal-to-noise ratio

### Assay validation and sample stability

The results of matrix effect are summarized in Table S2. As the matrix factor was detected to be approximately 1, this method did not appear to be influenced by urine matrix.

Evaluated values of recovery, precision, calibration curve linearity, and sensitivity are shown in Table 2. Absolute recovery rates of analytes ranged from 74% for DEET to 88% for icaridin. Loss of target compounds occurred during the SPE procedure, mainly the washing process with 25% methanol. However, this wash procedure contributes to the low matrix effect in LC-MS/MS analysis. In the intraday and interday precision concentration at 0.5 µg/L, which is near LLOQ level, over 15% RSD was observed in DHMB. For all analytes, precision concentrations over 1 µg/L showed less than 15% RSD. Coefficients of determination (*R*^2^) at our designated concentration range were >0.999. The calibration curve of icaridin is shown in Fig. S1. These results indicate that the developed method is reproducible and shows acceptable accuracy and precision. LOD of urine was 0.06–0.11 µg/L, and the LLOQ was 0.21–0.37 µg/L. The results of the chromatogram of pooled urine spiked with skin-IR biomarkers at various concentration ranges (0.5–40 µg/L) are shown in Fig. S2.

The stability of exposure biomarkers was evaluated considering two storage temperatures and three freeze-thaw cycles using urine samples (Table S3). Although all analytes in the urine are stable at −80 °C storage condition, icaridin concentrations decreased slightly (up to 85% of freshly prepared sample) after one week of storage at room temperature or 4 °C. After subjecting the urine to three freeze-thaw cycles, the concentrations of analytes except for DHMB were 89–101% compared with those of samples without freeze-thaw cycles. DHMB concentration was reduced by 14% compared to that immediately after sample preparation. The SPE-prepared samples for icaridin were stable for at least two days (less than 10% change) at 0.5, 1, 3, 10, and 20 µg/L concentrations of the urine in an LC auto injector.

### Application of the developed method to urine samples

**Fig. 3:**
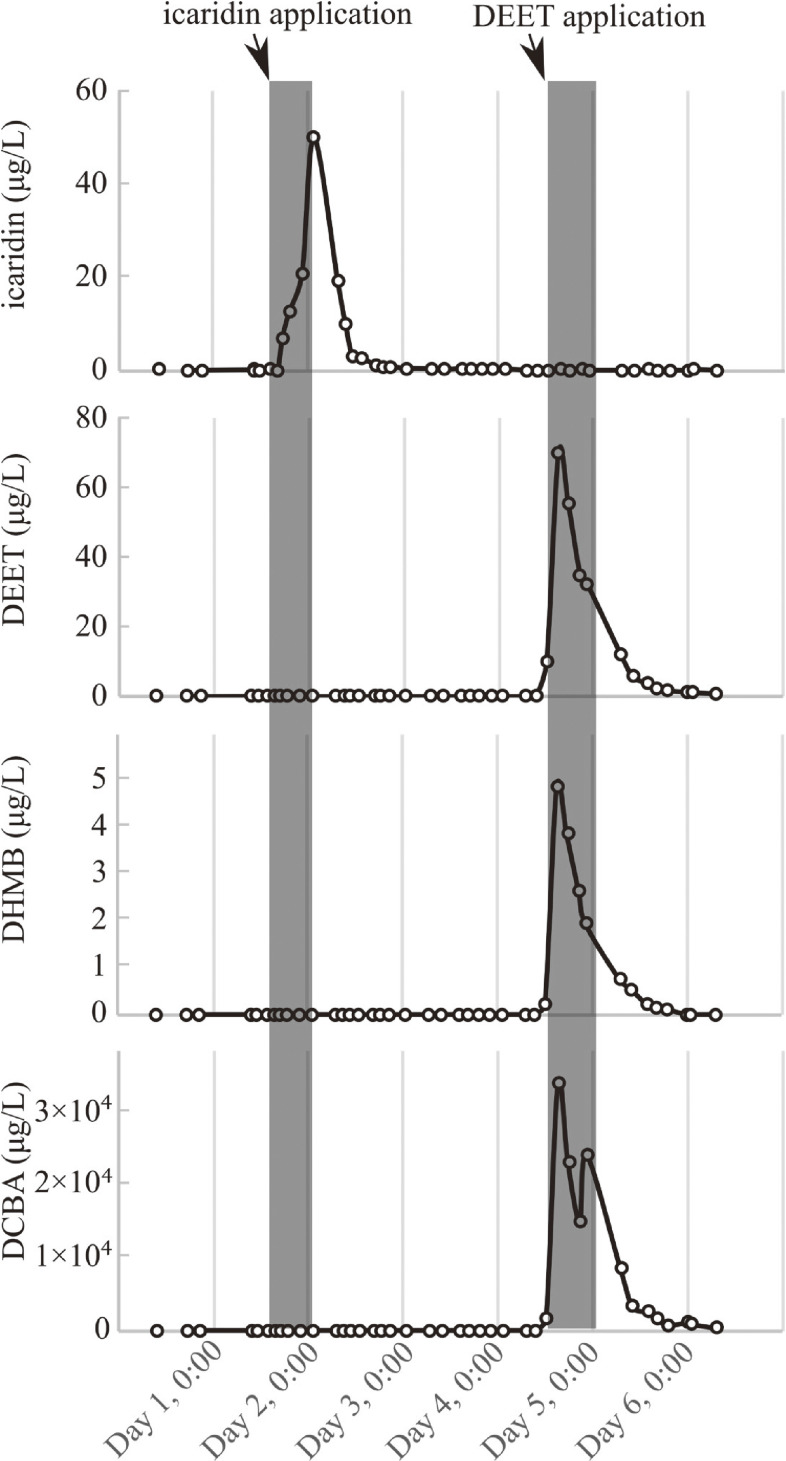
The concentration and time-courses of skin-IR exposure biomarkers in urine obtained from subject number 1. The gray bars represent the treatment period of the skin-IR, with the end of each bar indicating the time when the skin-IR was washed off with a shower.

The developed method was used to detect biomarkers in urine samples from five Japanese adults. Table 3 shows the characteristics of participants and the status of skin-IR usage. The maximum concentration of exposure biomarkers and the corresponding time after product use are listed in Table 4. The maximum concentration (50 µg/L) of icaridin was detected at 9.3 h after using an icaridin-containing product. The maximum concentration of urinary DEET (5.1–69.8 µg/L) was detected in four participants, from 4–14.8 h after the use of a DEET-containing product. DCBA in the urine reached concentrations >7000 µg/L, which is approximately two orders of magnitude higher than those of other exposure biomarkers. Figure 3 shows the concentration-time profile in the urine of Subject 1, who used both icaridin and DEET, as a representative example.

**Table 3:** Characteristics of the participants who used icaridin- or DEET-containing products.

**Participant number**	**Age**	**Gender**	**Ingredient of the product used**	**Number of times of using a product**
1	43	Male	Icaridin, DEET	one for each
2	21	Female	DEET	one
3	22	Male	DEET	one
4	20	Female	DEET	one
5	24	Female	DEET	four

**Table 4:** Exposure period (h) and the maximum concentration and time after initial use of skin-IRs.

**Subject number**	**Exposure period (h)**		***C*_max_, µg/L (*T*_max_, h)**			
	
	**Icaridin**	**DEET**	**Icaridin**	**DEET**	**DHMB**	**DCBA**
1	9.3	13.3	50.0 (9.3 h)	69.8 (4.0 h)	4.9 (4.0 h)	3.5 × 10^4^ (4.0 h)
2		5.5		24.3 (4.0 h)	1.1 (4.0 h)	1.0 × 10^4^ (4.0 h)
3		13.8		5.1 (14.8 h)	0.4 (14.8 h)	7.1 × 10^3^ (14.8 h)
4		3.3		<LOD	<LOD	38.8 (11.5 h)
5		12.5–18*		10.7 (12.1 h)	0.4 (12.1 h)	7.2 × 10^3^ (12.1 h)

*DEET was used four times by the subject in the urine collection days. The concentration data were obtained for the first application of DEET.

Urine samples with high concentrations of icaridin and DEET were selected for the deconjugation procedure. Upon deconjugation, urinary DCBA increased slightly, whereas DHMB concentration increased approximately 20- to 80-fold, as shown in Table S4. The deconjugation procedure did not change the concentrations of urinary icaridin and DEET.

The results of the inter-laboratory cross-validation study are summarized in Table S5. Statistical analyses, such as Bland-Altman analysis, were not performed due to the small sample size above LLOQ. The percent deviations were observed to range from 4% for icaridin (sample No. 5) and 34% for DHMB (sample No. 5). DCBA was successfully detected in SRM 3673 at 1.15 and 0.85 µg/L.

## Discussion

### Performance of the optimized method

To the best of our knowledge, this is the first study to quantify the icaridin levels in humans. In this study, we established a method for the simultaneous determination of urinary icaridin, DEET, and its metabolites as exposure biomarkers. Furthermore, our method successfully quantified icaridin and DEET exposure biomarkers in human urine in an observational study. Our novel method and information regarding exposure biomarkers, including stability and brief kinetics, may contribute to the further development of skin-IR risk assessments.

As the first step in our method, the sample preparation procedure and separation analysis were optimized for a highly sensitive output. The developed method was compared with five previously reported methods [25–30] (Table S6). Simple and typical SPE preparation procedures based on reversed-phase chromatography were adopted in all methods, including our newly developed method. SPE is advantageous compared to liquid-liquid extraction owing to the requirement of less solvent, short sample preparation time, and ease of operation and automation. The automation system in our SPE procedure took approximately 40 min to analyze 96 samples, indicating that high-throughput analysis may be performed using our method and applied in epidemiological studies. The sensitivity of this method for DEET and its metabolites was approximately the same or slightly superior to that of the other study methods. One advantage of this study is that icaridin can be measured simultaneously with other skin-IRs.

Slightly low absolute recovery rates were observed (74–88%). Based on the U.S. Food and Drug Administration for Bioanalytical Method Validation Guidance for Industry, the results of the intraday and interday precisions were satisfactory (<20%) for all concentration ranges of all exposure biomarkers. One limitation of this study is that isotope-labeled DEET metabolites were not utilized. Although isotope-labeled DEET metabolites are commercially unavailable, further improvement using the isotope-labeled I.S. might achieve higher precision for DHMB and DCBA.

This study is the first to report the storage and freeze-thaw cycle stability of these skin-IR exposure biomarkers in human urine, indicating that residual urine samples obtained from other studies could be used for future human biomonitoring (HBM) studies regarding icaridin and DEET exposure. However, the effect of biodegradation on the stability of exposure biomarkers requires further investigation. Thus, urine samples should be handled carefully to avoid bacterial proliferation in urine samples.

Currently, limited data are available regarding the toxicokinetics of icaridin. After dermal application of radiolabeled icaridin at a low dose in rats, approximately 60% was absorbed and 73–88% was excreted in the urine [31]. In a human dermal absorption study, approximately 94% of the absorbed radiolabeled icaridin was recovered in the urine within 24 h of the initial administration [32]. These data were obtained from radioactivity monitoring, reflecting only the sum of icaridin and its metabolite excretion levels. A total of 19 metabolites have been identified in the urine and feces of both intravenously injected and dermally dosed rats [11]. The best biomarkers for icaridin exposure assessment have not yet been identified. Based on an experimental study, the predominant modifications of icaridin (parent compound) were phase 1 reactions in which the piperidine ring or the 2-methylpropyl sidechain was hydroxylated or the hydroxyethyl sidechain was oxidized to the carbonyl moiety [17]. Metabolite standards are currently very difficult to obtain. Nonetheless, the identification of optimal candidate biomarkers for icaridin exposure remains crucial for advancing future research efforts. Moreover, further studies are required to elucidate the relationship between icaridin exposure and biomarkers in human urine.

DEET is metabolized via ring methyl oxidation and *N*-deethylation, which is mediated by cytochrome P450 (e.g., CYP2B6 and 3A4) [33]. Urine is the principal route of excretion of radioactivity after radiolabeled DEET administration and accounts for approximately 7% of the applied dose [7]. As shown in Table S6, urinary DEET and its metabolites were monitored as exposure biomarkers for DEET. The urinary metabolite profile has also been reported to include glucuronide conjugates associated with phase II metabolism [34]. Some previously reported measurement methods include a deconjugation process [24–26]. The concentration of DHMB increased significantly after deconjugation, but that of DCBA, DEET, and icaridin did not increase in our preliminary study (Table S5). Therefore, the deconjugation process was omitted from our method to avoid additional costs and labor. Further studies are needed to clarify the implications of the measurement of conjugate forms of DHMB and DCBA in the urine.

### Application of the developed method to urine samples from icaridin and DEET users

Icaridin, DEET, and its metabolites were successfully detected in urine samples obtained from Japanese adults who used skin-IRs prior to the experiments. In addition, this is the first report of icaridin quantification in human specimens for epidemiological applications. Furthermore, it is worth noting that the time of maximum urinary concentration of exposure biomarkers after icaridin or DEET application was observed in human samples.

Five participants used icaridin or DEET to repel biting, pests such as mosquitoes and ticks. One participant (Subject 1) used products containing icaridin and DEET on different days within a week. Urinary icaridin concentrations reached their maximum level 9 h after the initial use, which was two or more times later than the time at which the urinary DEET concentration reached its maximum. Since no information was available regarding the amount of icaridin applied in Subject 1, any calculation such as urinary excretion rate relative to icaridin application amount was adopted. Subject 4 reported using DEET; however, the levels of exposure markers in urine were lower than those in the other participants. This difference is likely attributable to variations in the amount and duration of DEET application, as well as differences in metabolic capacity. Unfortunately, the lack of information on the quantity of skin-IR applied, the site on the body where it was applied, and the solvent of skin-IR is a limitation of this study. In addition, the relevant absorption, distribution, metabolism, and excretion values should be investigated in the future. It should be noted that our monitoring data strongly suggest that this method may be applicable to HBM studies with participants exposed to skin-IR.

The preliminary inter-laboratory cross-validation study, which obtained comparable measurement data from two independent laboratories, supports the robustness of our present method, despite the limited number of urine samples.

## Conclusions

In conclusion, the developed off-line SPE LC-MS/MS method was successfully applied to evaluate exposure biomarkers of skin-IR in urine samples of Japanese adults. A recent study suggested a positive correlation between DEET exposure and obesity [18]; thus, epidemiological studies are important for revealing the unknown adverse health effects of icaridin and DEET exposure. Our method can make a significant contribution to future HBM exposure assessments.

## Abbreviations

skin-IR: skin-insect repellents
SPE: solid-phase extraction
LC-MS/MS: high-performance liquid chromatography-tandem mass spectrometry
DHMB: *N*,*N*-diethyl-3-(hydroxymethyl)benzamide
DCBA: 3-(diethylcarbamoyl) benzoic acid
HETP: height equivalent of one theoretical plate
LOD: limit of detection
LLOQ: lower limit of quantification
RSD: relative standard deviation

## Supplementary information

**Additional file 1:** **Table S1** HETP of seven LC columns. **Table S2** Absolute and relative matrix factors. **Table S3** Icaridin, DEET, DHMB, and DCBA stabilities in urine. **Table S4** Concentrations of DHMB and DCBA detected after or without the deconjugation procedure. **Table S5** Urinary concentration of skin-insect repellent biomarkers in measured by Lab. A and Lab. B. **Table S6** Comparative analyses of the present study with previous reports on DEET exposure marker determination. **Fig. S1** Calibration curve of icaridin at concentrations of 0.5–40 µg/L. **Fig. S2** Mass chromatograms of urinary icaridin, DEET, DHMB, and DCBA concentrations at 0.5–40 µg/L.

## Declarations

### Availability of data and material

Data cannot be shared for privacy or ethical reasons.
