# Supplementary material for: Determination of skin-insect repellent icaridin and DEET in human urine using solid-phase extraction and liquid chromatography with tandem mass spectrometry and its application to a sample of Japanese adults
Source: Environ Health Prev Med. 2025 Mar 20;30:18. doi: 10.1265/ehpm.24-00220 (PMC11955798; doi:10.1265/ehpm.24-00220)
Supplement: Supplementary file 1 — Additional file 1: Table S1 HETP of seven LC columns. Table S2 Absolute and relative matrix factors. Table S3 Icaridin, DEET, DHMB, and DCBA stabilities in urine. Table S4 Concentrations of DHMB and DCBA detected after or without the deconjugation procedure. Table S5 Urinary concentration of skin-insect repellent biomarkers in measured by Lab. A and Lab. B. Table S6 Comparative analyses of the present study with previous reports on DEET exposure marker determination. Fig. S1 Calibration curve of icaridin at concentrations of 0.5–40 µg/L. Fig. S2 Mass chromatograms of urinary icaridin, DEET, DHMB, and DCBA concentrations at 0.5–40 µg/L. [file ehpm-30-018-s001.docx]

Supplementary Material

Determination of skin-insect repellent icaridin and DEET in human urine using solid-phase extraction and liquid chromatography with tandem mass spectrometry and its application to a sample of Japanese adults

Nanami NISHIHARA^1^ • Tomohiko ISOBE^2^ • Mai TAKAGI^2^ • Toshiki TAJIMA^1^• Yugo KITAHARA^1^• Mai HAYASHI^1^ • Isao SAITO^1^ • Satoru WATANABE^1^ • Miyuki IWAI-SHIMADA^2^ • Jun UEYAMA^1*^

*** Correspondence:** Jun Ueyama: ueyama@met.nagoya-u.ac.jp

| **Table S1** HETP of seven LC columns | | | | |
| --- | --- | --- | --- | --- |
| LC-column name | HETP | | | |
|  | Icaridin | DEET | DHMB | DCBA |
| InertSustain^TM^ AQ-C18 | 0.14 | 0.13 | 3.11 | 0.43 |
| Poroshell 120 EC-C18 | 0.27 | 0.26 |  | 0.58 |
| InfinityLab Poroshell 120 CS-C18 | 0.51 | 0.85 | 2.54 | 2.17 |
| ZORBAX SB-C18 | 0.21 | 0.15 |  |  |
| CAPCELL PAK C18 | 0.25 | 0.24 | 14.65 | 1.43 |
| CAPCELL CORE ADME | 0.28 | 0.12 | 3.83 | 0.86 |
| CAPCELL CORE PFP | 0.35 | 0.26 | 10.30 | 2.68 |
| Cadenza CD-C18 | 0.20 | 0.19 | 3.89 | 0.95 |
| HETP, height equivalent of one theoretical plate. Blanks indicate unavailable data. | | | | |

| **Table S2** Absolute and relative matrix factors. | | | | | | | |  |
| --- | --- | --- | --- | --- | --- | --- | --- | --- |
| Concentration  (µg/L) | Absolute matrix factors  (comparative peak area) | | | | Relative matrix factors  (comparative I.S. ratio) | | | |
|  | Icaridin | DEET | DHMB | DCBA | Icaridin | DEET | DHMB | DCBA |
| 1 | 0.91 | 0.95 | 0.92 | 1.19 | 0.96 | 1.14 | 1.10 | 1.41 |
| 10 | 0.98 | 0.93 | 1.10 | 1.37 | 0.91 | 0.94 | 1.10 | 1.37 |
| 40 | 1.07 | 1.04 | 1.05 | 1.18 | 0.96 | 1.02 | 1.02 | 1.15 |
| 100 | 0.97 | 0.90 | 1.03 | 1.12 | 1.01 | 1.01 | 1.16 | 1.26 |
| 1000 |  |  |  | 1.02 |  |  |  | 1.08 |
| 10000 |  |  |  | 1.11 |  |  |  | 1.05 |

| **Table S3** Icaridin, DEET, DHMB, and DCBA stabilities in urine. | | | | | | | | | |  |
| --- | --- | --- | --- | --- | --- | --- | --- | --- | --- | --- |
|  |  | Concentration (µg/L urine) | *n* | Storage  period | Results | | | | | |
|  |  |  |  |  | Icaridin | | DEET | | DHMB | DCBA |
| Prepared sample stability (%) | |  |  |  |  | |  | |  |  |
| (mean±RSD) | | 0.5  1  3  10  20 | 3  3  3  3  3 | 2 days  2 days  2 days  2 days  2 days | 91±4  92±6  90±6  98±3  100±1 | | 103±8  112±5  100±5  102±2  100±2 | | 104±9  92±5  96±3  100±2  102±3 | 102±10  91±4  102±1  107±2  106±3 |
| Freeze-thaw stability (%) | | 0.5 | 4 |  | 98±4 | | 101±5 | | 98±10 | 94±8 |
| (mean±RSD) | | 3  20 | 4  4 |  | 101±7  99±6 | | 100±3  99±2 | | 86±4  100±6 | 89±5  99±5 |
| Storage stability (%) | |  |  |  |  | |  | |  |  |
|  | 25°C | 0.5  3  20 | 2  2  2 | 1 week  1 week  1 week | 89  98  102 | | 98  95  97 | | 92  107  99 | 108  107  100 |
|  | 4°C | 0.5  3  20 | 2  2  2 | 1 week  1 week  1 week | 85  96  101 | | 88  100  95 | | 94  97  93 | 102  101  94 |
| N, number of observations; RSD, relative standard deviation | | | | | |  | |  | | |

| **Table S4** Concentrations of DHMB and DCBA detected after or without the deconjugation procedure. | | | | |
| --- | --- | --- | --- | --- |
| Deconjugation  procedure | | Sample ID | Concentrations (µg/L) | |
|  |  |  | DHMB | DCBA |
| No | | 1  2  3 | 2  50  37 | 6,945  12,790  1,119 |
| Yes | | 1  2  3 | 167  1,204  695 | 10,160  15,544  1,697 |
|  |  | | | |
|  |  | | | |

| **Table S5** Urinary concentration of skin-insect repellent biomarkers in measured by Lab. A and Lab. B. | | | | | | | | | | | | |  |
| --- | --- | --- | --- | --- | --- | --- | --- | --- | --- | --- | --- | --- | --- |
| Sample No. |  | Icaridin | |  | DEET | |  | DHMB | |  | DCBA | | |
|  |  | Lab. A | Lab. B |  | Lab. A | Lab. B |  | Lab. A | Lab. B |  | Lab. A | Lab. B | |
| 1 |  | - | - |  | - | - |  | - | - |  | 4.34 | 5.86 | |
| 2 |  | - | - |  | - | - |  | - | - |  | 0.68 | 0.76 | |
| 3 |  | - | - |  | - | - |  | - | - |  | - | - | |
| 4 |  | - | - |  | - | - |  | - | - |  | - | - | |
| 5 |  | 5.30 | 5.09 |  | 54.3 | 51.7 |  | 0.52 | 0.73 |  | 1.12 | 1.31 | |
| 6 (SRM 3673) |  | - | - |  | - | - |  | - | - |  | 1.15 | 0.85 | |
| SRM, Standard Reference Material; Lab. A, chemical analysis company located in Osaka prefecture; Lab. B, Nagoya University, which developed the measurement method.  A hyphen indicates that it is below the lower limit of quantification for present study. | | | | | | | | | | | | |  |

| Table S6 Comparative analyses of the present study with previous reports on DEET exposure marker determination. | | | | | | |
| --- | --- | --- | --- | --- | --- | --- |
| References  year | **Fišerová et al.**  **2021 (28)** | **Baker et al.**  **2019 (29)** | **Behniwal et al.**  **2017 (30)** | **Kuklenyik et al.**  **2013 (25)** | **Olsson et al.**  **2004 (26)** | **Present method** |
| Analytical apparatus | LC-MS/MS (ESI) | LC-MS/MS (ESI) | LC-MS/MS (ESI) | LC-MS/MS (APCI) | LC-MS/MS (APCI) | LC-MS/MS (ESI) |
| Mass spectrometer | AB Sciex QTrap 5500  (Sciex) | Vantage TSQ  (Thermo Fisher Scientific) | AB Sciex 5500  (Sciex) | TSQ Quantum Ultra™  (Thermo Fisher Scientific) | TSQ 7000  (Thermo Fisher Scientific) | 6430  (Agilent) |
| Analytical column | Acquity UPLC BEH C18  100 mm × 2.1 mm i.d. 1.7 µm (Waters) | Hypersil Gold aQ  150 mm × 4.6 mm i.d. 3 µm  (Thermo Fisher Scientific) | ACE Excel 2 C18-PFP  100 mm × 2.1 mm i.d. 3 µm  (Tomsic) | Prodigy 5µm Phenyl-3 (PH-3) 100 mm × 4.6 mm i.d. 5 µm  (Phenomenex) | Betasil phenyl column  100 mm × 4.6 mm i.d. 5 µm  (Keystone Scientific) | InertSustain AQ-C18  150 mm × 2.1 mm i.d. 3 µm  (GLSciences) |
| Sample preparation | Solid-phase extraction  (Oasis HLB) | Solid-phase extraction  (Chromolith Flash RP-18e) | Solid-phase extraction  (Oasis HLB) | Solid-phase extraction  (Chromolith Flash RP-18e) | Solid-phase extraction  (Oasis HLB) | Solid-phase extraction  (Evolute Express ABN) |
| Internal Standard | DEET-d6 | ECBA-d5, DCBA-d10 | DEET-d6 | DEET-d10, DHMB-d10,  DCBA-d10 | DEET-d6 | DEET-d10 |
| Needed urine volume (µL) | 500 | 200 | 1000 | 100 | 2000 | 500 |
| Analytes | DEET | ECBA, DCBA | DEET | DEET, DHMB, DCBA | DEET | DEET, DHMB, DCBA |
| LLOQ (µg/L) | 0.08 |  |  |  |  | 0.21 (DEET),  0.33 (DHMB),  0.37 (DCBA) |
| LOD (µg/L) | 0.03 | 0.05 (ECBA), 0.06 (DCBA) | 0.05 | 0.1 (DEET, DHMB)  1.0 (DCBA) | 0.1 | 0.06 (DEET),  0.10 (DHMB),  0.11 (DCBA) |
| ECBA, 3-ethyl-carbamoyl benzoic acid | | | | | | |
|  | | | | | | |

**Figure S1**


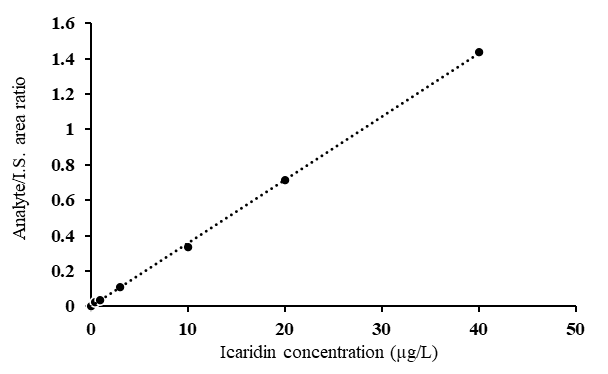


Fig. S1 Calibration curve of icaridin at concentrations of 0.5–40 µg/L.

**Figure S2**

**icaridin　　　　　　　　　　　　　　DEET**


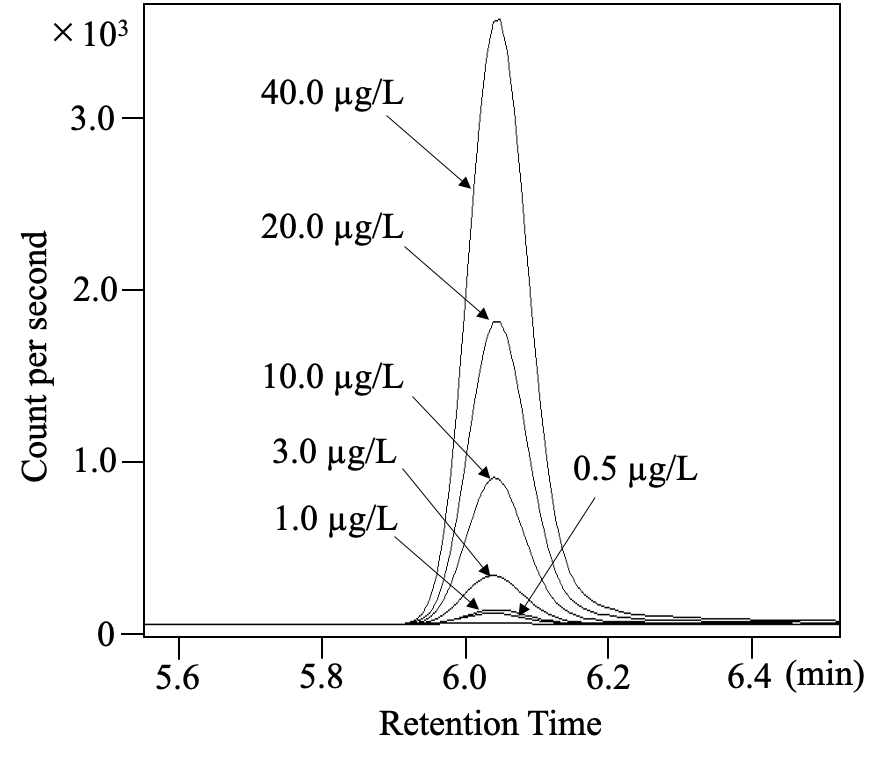
　　
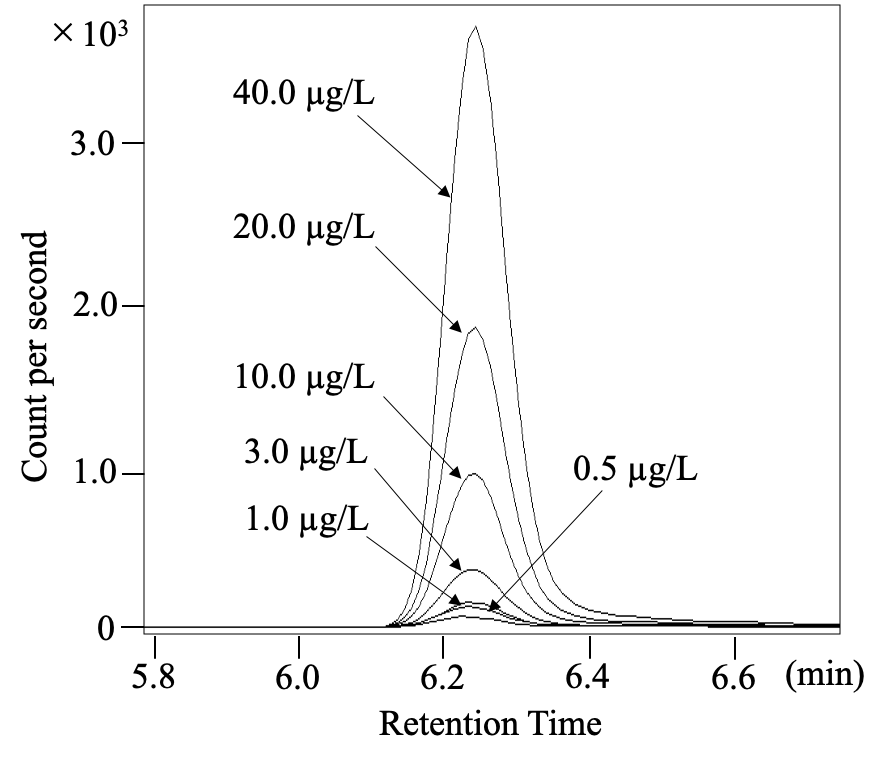


**DHMB　　　　　　　　　　　　　　　　　　　　　　　DCBA**


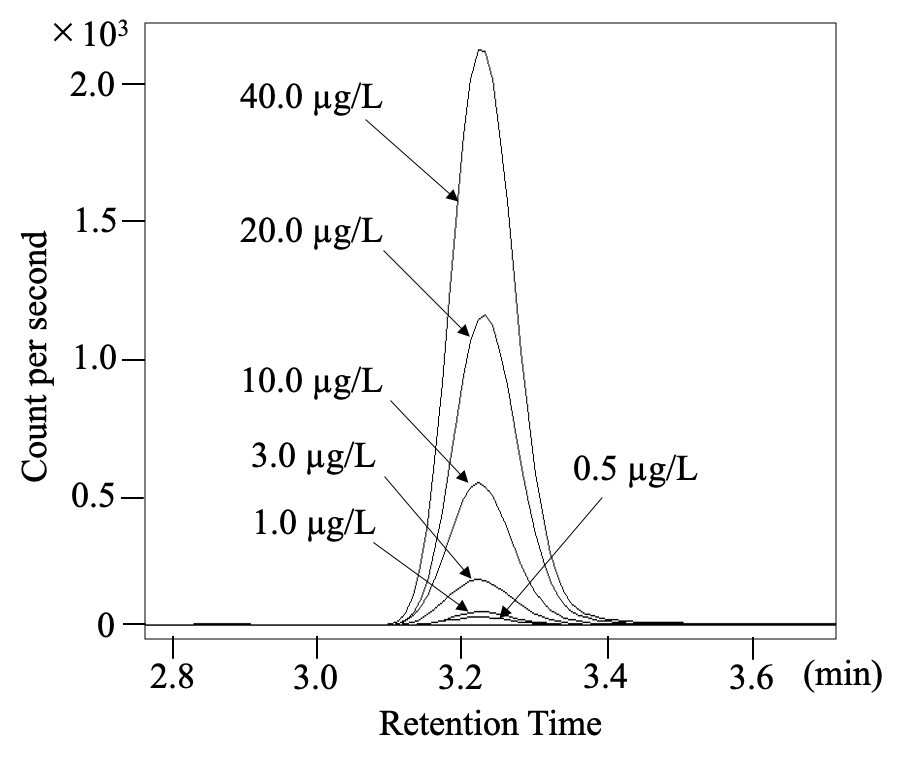

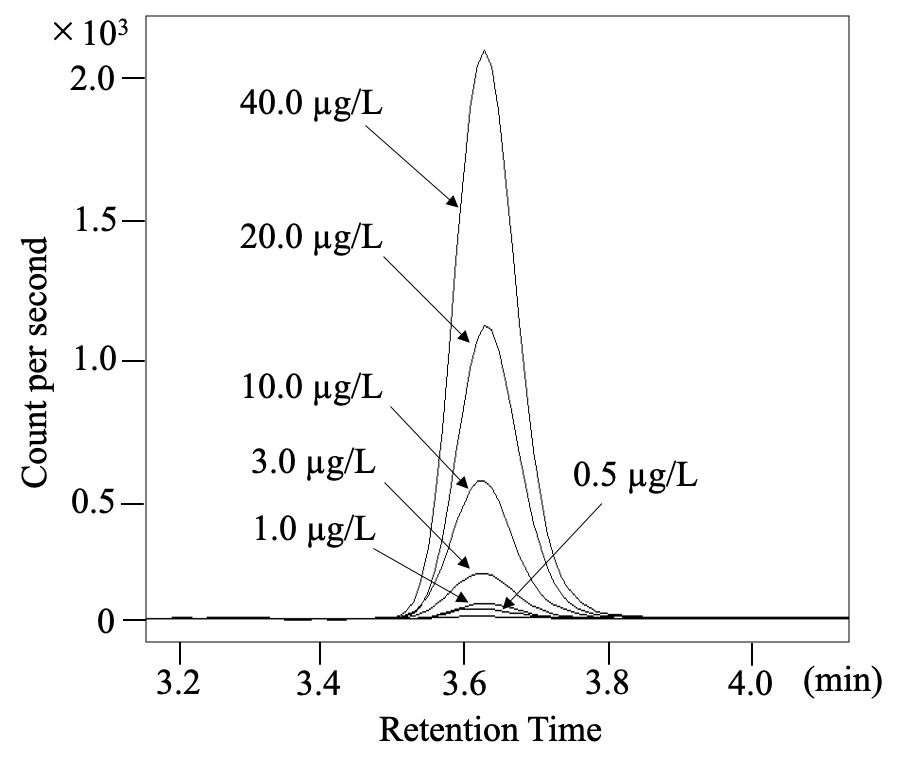


Fig. S2 Mass chromatograms of urinary icaridin, DEET, DHMB, and DCBA concentrations at 0.5–40 µg/L.
